# Integration of a Decrescent Transcriptome and Metabolomics Dataset of *Peucedanum praeruptorum* to Investigate the CYP450 and MDR Genes Involved in Coumarins Biosynthesis and Transport

**DOI:** 10.3389/fpls.2015.00996

**Published:** 2015-12-10

**Authors:** Yucheng Zhao, Tingting Liu, Jun Luo, Qian Zhang, Sheng Xu, Chao Han, Jinfang Xu, Menghan Chen, Yijun Chen, Lingyi Kong

**Affiliations:** ^1^State Key Laboratory of Natural Medicines, Department of Natural Medicinal Chemistry, China Pharmaceutical UniversityNanjing, China; ^2^Institute of Botany, Jiangsu Province and Chinese Academy of SciencesNanjing, China

**Keywords:** *P. praeruptorum*, transcriptomic, metabolomics, coumarins, biosynthesis, transporter

## Abstract

*Peucedanum praeruptorum* Dunn is well-known traditional Chinese medicine. However, little is known in the biosynthesis and the transport mechanisms of its coumarin compounds at the molecular level. Although transcriptomic sequence is playing an increasingly significant role in gene discovery, it is not sufficient in predicting the specific function of target gene. Furthermore, there is also a huge database to be analyzed. In this study, RNA sequencing assisted transcriptome dataset and high-performance liquid chromatography (HPLC) coupled with electrospray-ionization quadrupole time-of-flight mass spectrometry (Q-TOF MS)-based metabolomics dataset of *P. praeruptorum* were firstly constructed for gene discovery and compound identification. Subsequently, methyl jasmonate (MeJA)-induced gene expression analysis and metabolomics analysis were conducted to narrow-down the dataset for selecting the candidate genes and the potential marker metabolites. Finally, the genes involved in coumarins biosynthesis and transport were predicted with parallel analysis of transcript and metabolic profiles. As a result, a total of 40,952 unigenes and 19 coumarin compounds were obtained. Based on the results of gene expression and metabolomics analysis, 7 cytochrome-P450 and 8 multidrug resistance transporter unigenes were selected as candidate genes and 8 marker compounds were selected as biomarkers, respectively. The parallel analysis of gene expression and metabolites accumulation indicated that the gene labeled as *23,746, 228*, and *30,922* were related to the formation of the coumarin core compounds whereas *36,276* and *9533* participated in the prenylation, hydroxylation, cyclization or structural modification. Similarly, *1462, 20,815*, and *15,318* participated in the transport of coumarin core compounds while 124,029 and 324,293 participated in the transport of the modified compounds. This finding suggested that integration of a decrescent transcriptome and metabolomics dataset could largely narrow down the number of gene to be investigated and significantly improve the efficiency of functional gene predication. In addition, the large amount of transcriptomic data produced from *P. praeruptorum* and the genes discovered in this study would provide useful information in investigating the biosynthesis and transport mechanism of coumarins.

## Introduction

**Figure 1:**
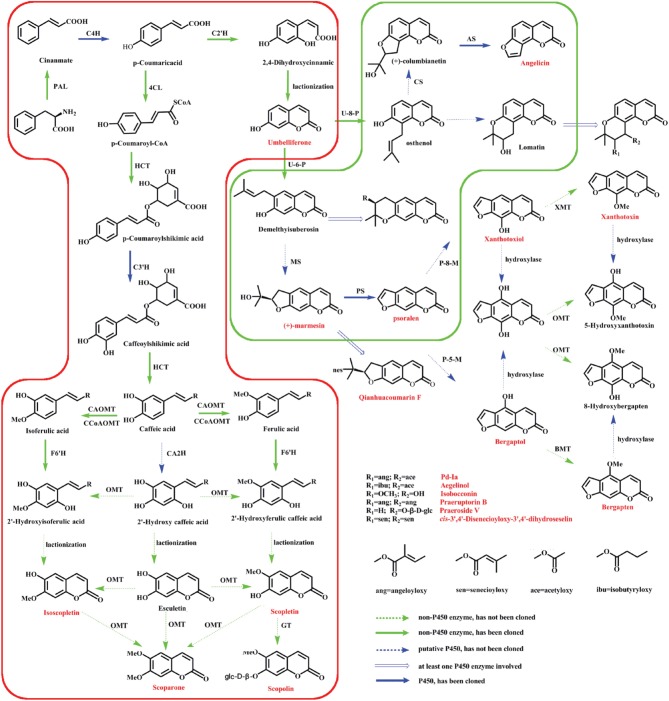
**Putative coumarins biosynthetic pathway in *P. praeruptorum*.** The three stages of proposed biosynthetic pathways: formation of coumarins core (red box), prenylation, hydroxylation, and cyclization (green box) and structure modifications. PAL, phenylalanine ammonia lyase; C4H, cinnamate 4-hydroxylase; 4CL, 4-coumarate: coenzyme A ligase; HCT, hydroxycinnamoyl CoA shikimate/quinate hydroxycinnamoyl transferase; CCoAOMT, caffeoyl-CoA O-methyltransferase; C3H, p-coumarate 3-hydroxylase; CA2H, caffeic acid 2-hydroxylase; CAOMT, caffeic acid O-methyltransferase; O-MT, O-methyl-transferase; GT, glycosyltransferase; C2'H, Cinnamic acid 2′-hydroxylase; F6'H, feruloyl-CoA 6′-hydroxylase; U-6-P, Umbelliferone 6-prenyltransferase; U-8-P, Umbelliferone 8-prenyltransferase; MS, Marmesin synthase; PS, Psoralen synthase; P-8-M, Psoralen 8-monoooxgenase; P-5-M, Psoralen 5-monoooxgenase; XMT, Xanthotoxiol O-methyltransferase; BMT, Bergaptol O-methyltransferase; CS, Columbianetin synthase; AS, Angelicin synthase. The compounds identified in *P. praeruptorum* are marked with red characters.

Radix Peucedani (Baihua Qianhu in Chinese), the roots of *Peucedanum praeruptorum* Dunn, is one of the most popular traditional Chinese medicine and has been used for more than 1500 years. It is also listed in the current Pharmacopeia of the Peoples' Republic of China (Commission, 2010). Traditionally, Radix Peucedani is used as a kind of herbal medicine for reducing fevers and resolving phlegm and is generally employed to treat anemopyretic cold, cough with abundant phlegm and congested chest (Zhou et al., 2014). Modern pharmacological studies have indicated that the extracts of *P. praeruptorum* (the main chemical constituents are coumarins) also displayed anti-cancer, anti-inflammatory, anti-hyperglycemic, anti-oxidant, and calcium-channel-blocking properties (Wu et al., 2003; Kumar et al., 2009; Yu et al., 2012). However, little is known in coumarins biosynthetic pathways, even at the biochemical level (Bourgaud et al., 2006). As shown in Figure 1, among the three stages of proposed biosynthetic pathways, only the enzyme involved in the first stage are thoroughly studied (Gaid et al., 2012; Karamat et al., 2012; Kim et al., 2012). It is well known that secondary metabolites can accumulate at a high concentration in particular tissues. In addition, the roots of *P. praeruptorum* are usually chosen in medical treatments (Yazaki et al., 2008; Commission, 2010). While, other questions, such as how this kind of compound accumulates and how they maintain at an appropriate concentration in different compartments, arose. Recently, a mountain of reports indicated that a transporter-based transport mechanism was involved in the process of accumulation. In addition, some transporters had also been identified. However, there are no reports on the transport mechanisms of coumarin compounds. Even worse, no coumarins transporter was identified (Li et al., 2002; Yazaki, 2005, 2006; Rea, 2007; Mehrshahi et al., 2013; Yu and De Luca, 2013). Considering their important pharmacological benefits and the uncertainties in coumarins biosynthesis and transport, it is critical to investigate the enzymes and genes relevant to their biosynthesis and transport.

As an example for gene function prediction, “guilt-by-association” or “comparative co-expression” has emerged in the past 10 years and displayed powerful performance (Usadel et al., 2009; Mutwil et al., 2011; Movahedi et al., 2012). However, it seems difficult in predicting gene that has not been reported, especially the gene that no reference conserved co-expression clusters (Movahedi et al., 2012). Another success in studying functional genomics is integrating analysis of comprehensive gene expression and metabolic profiling according to the fact that the enhanced accumulation of metabolites was preceded by coordinated increases in the transcript level of relevant genes (Saito et al., 2008; Saito and Matsuda, 2010; Choi et al., 2012; Gaid et al., 2012). However, these successes depend largely on the availability of genome sequence data and metabolite accumulation database (Saito et al., 2008; Saito and Matsuda, 2010). Hence, it is advantageous to study the functional genomics of model plants such as *Arabidopsis* and rice which have genome data and metabolite database (Saito et al., 2008; Yonekura-Sakakibara et al., 2012), but it is not necessarily straightforward in non-model plants especially *P. praeruptorum* whose transcriptome data is still unknown. As an important supplement to genomic analysis, transcriptomic analysis is playing an increasingly significant role in the discovery of new genes involved in plant secondary metabolism, particularly in the case of plants for which the full genomic sequences are not currently available. Next-generation-sequencing technology (NGS), such as Roche/454 and Illumina HiSeq platforms, has emerged as a primary tool for high-through-put sequencing which can dramatically improve the efficiency and rapidity of gene discovery (Schuster, 2008; Ansorge, 2009; Wang et al., 2014). However, it is insufficient in gene discovery just using sequence data.

**Figure 2:**
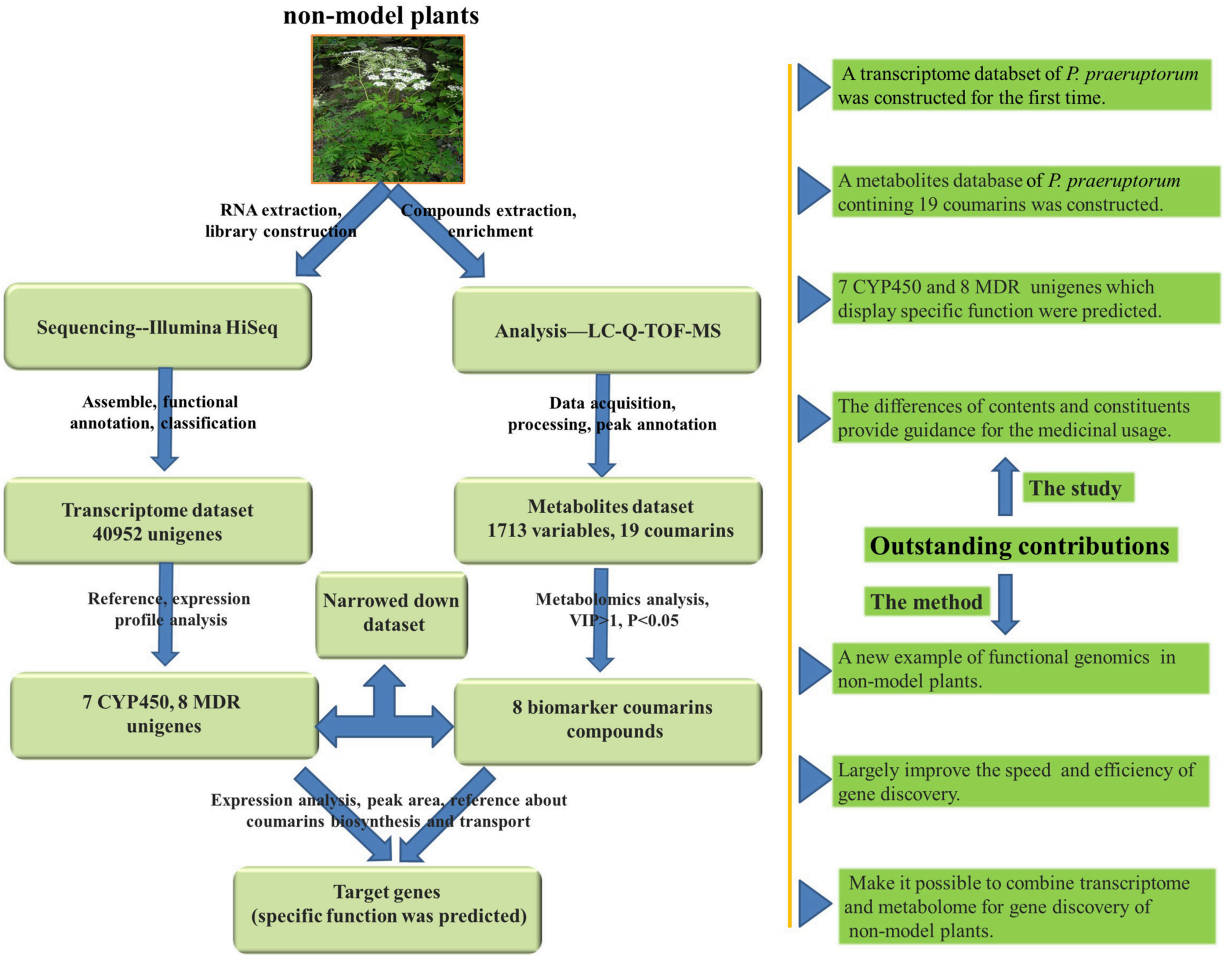
**An overview of the experimental design and outstanding contributions**. The non-model medical plant was used to transcriptome and metabolomics analysis. At first, the plant materials were prepared for sequencing and chemical analysis with Illumina Hiseq and LC-Q-TOF-MS. Secondly, after data acquisition, processing, assembly, and annotation, the transcriptome and metabolomics dataset was constructed. Thirdly, according to the speculative coumarins biosynthesis and transport mechanism and the previous reports, a decrescent transcriptome and metabolomics dataset were re-constructed with MeJA-induced gene expression analysis and metabolomics analysis. Finally, the parallel analysis of gene expression and metabolomics accumulation was used to identify the gene involved in coumarins biosynthesis and transport. The outstanding contributions are also listed in the right side and the pictures used in this figure are download form http://image.baidu.com/.

It is generally acceptable and evaluated the assumption of correlation between gene expression and metabolite accumulation and plenty of works have been reported (Hirai et al., 2007; Saito et al., 2008). However, unlike *Arabidopsis*, there is no metabolite database available in *P. praeruptorum* (Saito et al., 2008). Considering the complicated chemical constituents of coumarins in *P. praeruptorum*, methods need to be established to analyze and construct the metabolite dataset. Hence, in this study, HPLC coupled with Q-TOF MS was used to identify the metabolites because it is rapid and can provide accurate mass measurements (Ling et al., 2013). Merging with transcriptome dataset, it could largely improve the rapidity, accuracy and efficiency of gene discovery (Yamazaki et al., 2013; Li et al., 2015). However, correlation analysis of the whole transcriptomic and metabolomics seems intertwined and unnecessary because we mainly concern some certain pathways or genes. Thus, in this study, a decrescent dataset of transcriptome and metabolomics of *P. praeruptorum* was constructed to investigate the target gene involved in our specific pathway. As described in Figure 2, the transcriptome sequencing and Q-TOF-MS/MS-based metabolites analysis were first conducted to construct the dataset. Then, expression analysis and metabolomics analysis were conducted to establish the descendant dataset. Finally, the differences in the peak intensities and distributions of the biomarker compounds in the different tissues were analyzed in conjunction with the expression analysis of selected gene to identify the genes involved in the biosynthesis and transport of coumarins.

As a result, putative CYP450 and MDR genes that participated in the biosynthesis and transport of coumarin compounds were predicted at the gene and metabolic level. Additionally, the specific accumulation of coumarin compounds in the roots, stems, and leaves was also investigated, the results of which may facilitate the utilization of portions of medicinal plants. To the best of our knowledge, this is the first study to examine the transcriptome of *P. praeruptorum* and the first attempt to merge transcriptomic and metabolomics to understand the biosynthetic pathways and identify the genes that participate in the biosynthesis and transport of coumarins.

## Materials and methods

### Plant materials and RNA preparation

The two-year-old *P. praeruptorum* material was collected from the fields of Ningguo City, Anhui Province, China, which has the reputation of being “The County of Qianhu in China.” After removing the soil and deadwood, the plants were immediately frozen in liquid nitrogen and were stored at −80°C until use for total RNA isolation. Plants were also transplanted into plastic basins containing with a mixture of vermiculite, perlite, and peat moss at a 1:1:1 ratio and were grown in an environmentally controlled chamber with a long photoperiod (16 h light and 8 h dark) at 25°C, 40–65% relative humidity and 3000 lux of light intensity until use in the MeJA induction experiment. The total RNA was isolated using TransZol Plant reagent (TransGen Biotech, Beijing, China) according to the manufacturer's recommendations, and the quantity and quality of the RNA were determined using a Spectramax plus384 enzyme-labeling instrument (Molecular Devices, Sunnyvale, USA) and 1% agarose gels. To evaluate the differences in tissue-specific expression and accomplish the aim of this study, the total RNA was extracted from a mixture of roots, stems and leaves for construction of a cDNA library. It was also used for tissue-specific expression analysis. All of the samples were treated with DNase I (Takara, Daliang, China) at a concentration of 1 unit/μg of total RNA.

### cDNA library construction and transcriptome sequence processing and assembly

A cDNA library was prepared with a kit provided by Illumina according to the manufacturer's recommendations and previously used methods (Yuan et al., 2015). For details, total RNA was extracted from a mixture of roots, stems and leaves using TransZol Plant reagent (TransGen Biotech, Beijing, China) according to the manufacturer's recommendations. Then, poly (A) mRNA was purified from the total RNA using oligo (dT) beads. After purification, mRNA was sheared into small pieces using fragmentation buffer. First-strand cDNA was annealed with random primers using cleaved mRNA fragments as templates. The second-strand cDNA was synthesized with DNA polymerase I and RNase H. Subsequently, the cDNA fragments were purified and ligated to index adapters. Finally, the cDNA library was constructed and subject to Illumina HiSeq 2500 system for high-throughput sequencing. The raw data were converted into fastaq format and then compressed as.gz files to be deposit to the National Center for Biotechnology Information (NCBI).

Sequence Read Achieve (SRA) sequence database under project accession number SRX997427. Due to the error rate in the raw data, low-quality-sequence fragments were removed via slip-window sampling using the following parameters: quality threshold of 20 (error rate = 1%), window size of 5 bp and length threshold of 35 bp. To adjust the pollution of the reads, 10^5^ sequences were randomly selected for sequence alignment of the nr reads at an *E*-value of < 1e^−10^ and a coverage level of >80%. After the pollution of the reads was cleaned, the good reads was used to assemble transcripts and unigenes using Trinity software (version trinityrnaseq_r2013-02-25) [(http://trinityrnaseq.sf.net)]. The unigenes representing the longest transcripts at each loci (comp^*^_c^*^_) were assembled using the Chrysalis cluster module of the Trinity program. To normalize the abundance of the transcripts, a k-mer value of 25 RPKM (reads per kb of an exon model per million mapped reads) (Wagner et al., 2012) was applied and defined in this way:

$$\text{RPKM}=\frac{\text{total exon reads}}{\text{mapped reads}(\text{millions})*\text{exon length}(\text{KB})}$$

### Functional annotation and classification

To find the most descriptive annotation for each transcript sequence, BLAST searches (Altschul et al., 1997) were conducted based on sequence similarities using a series of databases (Kanehisa et al., 2002; Dimmer et al., 2012), with the significance threshold set at an *e*-value of ≤ le^−5^. The functional categories of these unique sequences were analyzed using the Gene Ontology (GO: http://www.geneontology.org/) database, AGI codes and the TAIR GO slim program provided by TAIR (Lamesch et al., 2012). Pathway assignments were conducted based on the KEGG mapping results (Du et al., 2014) and enzyme commission (EC) numbers were assigned to the unique sequences. The KOG/COGs (clusters of orthologous groups) of the proteins were aligned to the entries in the EggNOG database to predict and classify the possible functions of the unigene products (http://www.ncbi.nlm.nih.gov/COG/).

### MeJA elicitation and preparation of samples for HPLC-Q-TOF-MS/MS analysis

The MeJA stock solution was prepared at 200 mM in ethanol and was filter-sterilized. For short period elicitation, plants were placed in deionized water, and then an aliquot of the stock MeJA solution was added to deionized water to yield a final concentration of 200 μM. For treatment, the MeJA solution with a final concentration was sprayed onto the leaves. The treated samples and the untreated control samples (to which an equivalent amount of ethanol was applied) were harvested for analysis at 1, 3, 6, 9, 12, and 24 h after elicitation. The treated samples were divided into three parts, the roots, stems and leaves, and then the samples were dried until reaching a constant weight. A 0.5 g dried sample was sequentially extracted three times using methanol, with ultrasonication, for 30 min at room temperature (3 × 4 ml). The three methanol extracts were combined, and the mixture was concentrated under reduced pressure conditions and then readjusted to a volume of 5 ml. To separate the high-abundance contents (such as praeruptorin A and praeruptorin C) from the low-abundance contents and then concentrate the low-abundance contents, a C18E cartridge was used. Before this separation was accomplished, the cartridge was equilibrated using 20 ml of 50% methanol, after which 0.25 ml (containing approximately 20 mg of compounds) of the volume-readjusted extract was loaded onto the cartridge and was allowed to adsorb for approximately 4 h. Then, 20 ml of 50% methanol was loaded for elution, and the eluate was evaporated to dryness using a stream of nitrogen gas. Finally, 20 ml of 90% methanol was used to elute the high-abundance contents. The residue of the 50% methanol eluate was suspended in 250 μl of methanol, and the 90% methanol eluate was suspended in 1 ml of methanol for further analysis.

### Quantitative real-time PCR analysis

To determine the expression level of putative CYP450s and MDR transcripts in *P. praeruptorum* after MeJA elicitation, quantitative real-time PCR analysis was performed using the SYBR Green PCR Master Mix (Vazyme, Nangjing, China) with LightCycler 480 instrument (Roche Molecular Biochemicals, Mannheim, Germany). Each reaction mixture contained 10 μl of 2 × SYBR Green Master Mix Reagent, 10 ng of cDNA and 4 μM of gene-specific primers in a total volume of 20 μl. The cycling conditions were as follows: 1 cycle of 95°C for 5 min, 40 cycles of 95°C for 10 s, and then 60°C for 30 s, followed by 1 cycle of 95°C for 15 s, 60°C for 60 s, and 95°C for 15 s. The mean value of three replicates was normalized with glyceraldehyde-3-phosphate dehydrogenase (GAPDH) (*comp23086_c0_seq1* in our transcriptome dataset). PCR amplification was performed using primers specific for the putative CYP450 and MDR transporter transcripts, which are listed in Table S1. Primer 5.0 software was used to design the primers. The relative expression levels were calculated by comparing the CTs (cycle thresholds) of the target genes with that of GAPDH using the 2^−ΔΔCT^ method (Pfaffl, 2001).

### HPLC-Q-TOF-MS/MS analysis

LC/electrospray ionization (ESI)-Q-TOF-MS/MS, applied in the positive ionization mode, was used to detect the coumarin compounds. The LC analysis of the selected coumarin compounds was conducted using an Agilent 1290 HPLC system (Agilent Technologies, Santa Clara, CA, USA) equipped with a binary pump, an online degasser, an auto plate sampler and a thermostatically controlled column compartment. Chromatographic separations were performed using an Agilent ZORBAX SB-C18 column (4.6 × 250 mm, 5 μm, Agilent Technologies, Santa Clara, CA, USA) with a solvent flow rate of 1 ml/min at 35°C. The sample injection volume was set to 10 μl, and the diode array detector was operated at 254 and 310 nm. The mobile phase A was 0.1% formic acid in water and the mobile phase B was methanol. The solvent gradient conditions A:B (v/v) are as follows: 0 min, 90:10; 3 min, 90:10; 7 min, 75:25; 8 min, 70:30; 12 min, 70:30; 13 min, 58:42; 20 min, 58:42; 21 min, 58:42; 25 min, 40:60; 28 min, 40:60; 35 min, 0:100; and 40 min, 0:100. A 5-min post-run equilibration to the initial mobile phase composition was conducted after each analysis was completed. This HPLC system was connected to a quadrupole time-of-flight mass spectrometer (Agilent Technologies, Santa Clara, CA, USA) that was equipped with an electrospray interface. The conditions of the ESI source were as follows: drying gas (N_2_) flow rate, 8.0 l/min; drying gas temperature, 300°C; nebulizer, 241 kPa (35 psig); capillary voltage, 4000 V; fragmentor voltage, 150 V; collision energy, 30 eV; skimmer voltage, 60 V, and octopole radio frequency, 250 V. This instrument provided a typical resolution of 9500 ± 500 (m/z 922.0098). All of the operations and analysis of data were controlled using Agilent LC-Q-TOF-MS Mass Hunter workstation software (version B.04.00).

### Data processing and metabolomics analysis

The Transomics metabolomics software package of the Waters Corporation was used for data processing because it could use the retention time (RT) and m/z data pairs as identifiers. The raw data regarding the coumarin content of the roots, stems and leaves were converted to common data format files using conversion software and then imported into the SIMCA-P13.0 software package (version 13.0, Umetrics, Umea, Sweden) for multivariate statistical analysis (Yan et al., 2015). As an unbiased statistical method, a principal component analysis (PCA) was first conducted to detect the inherent trends within the data, and then partial least-squares-discriminant analysis (PLS-DA) was conducted to study the variance in the levels in the different tissue compartments. To evaluate the quality of the model, the R^2^ and Q^2^ values were calculated. To select the potential biomarkers, the loading plot and variable importance in the project (VIP) values were first set at 1 (VIP >1), and then Student's *t*-test was conducted with the *P*-value set at 0.05 (*P* < 0.05) to determine the significantly different variables. The selected potential biomarkers were evaluated according to the metabolites identified based on their retention times, exact mass data and fragmentation ions. To analyze the gene expression and metabolites accumulation, three biological and technical replicates were used to obtain the data. Unless the special comments, the data were presented as mean of triplicate experiments ± standard deviation (SD). Graphs were generated using OriginPro 8 (OriginLab Corporation, Northampton, MA, USA).

In general, plant materials were cultured for RNA purification and MeJA treatment. A cDNA library was constructed for transcriptome sequencing and then the sequencing results were assembled, functional annotated and classified based on BLAST searches against public databases. The qPCR was used to analyze the gene expression. HPLC-Q-TOF-MS/MS was conducted for metabolomics analysis and investigating metabolites accumulation. Metabolomics analysis was used to identify the biomarkers.

## Results and discussion

### Transcriptomic analysis as a powerful tool for the discovery of target genes

**Figure 3:**
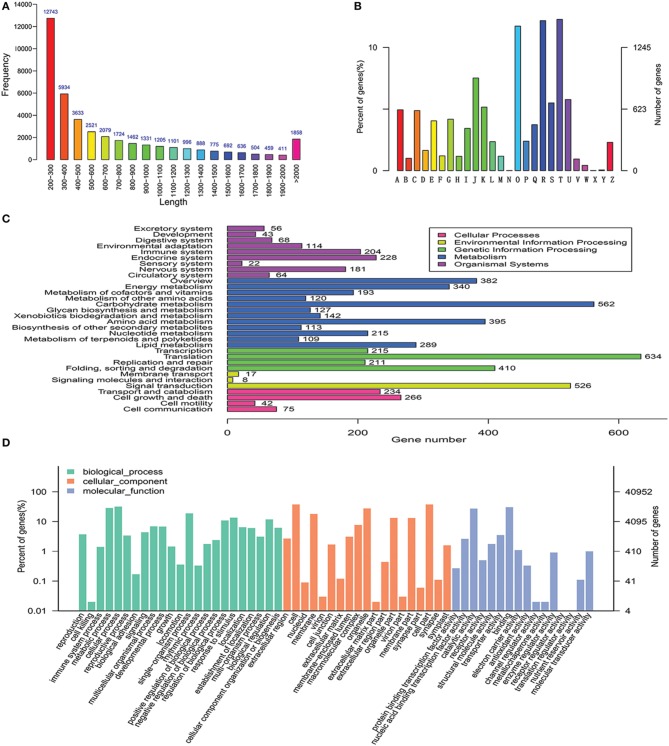
**De novo assembly, annotation and functional classification. (A)** Length distribution of the unigenes. **(B)** KOG classification assigned to unigenes. **(C)** KEGG classification of assembled unigenes. **(D)** Gene ontology classification of assembled unigenes. A, RNA processing and modification; B, Chromatin structure and dynamics; C, Energy production and conversion; D, Cell cycle control, cell division, chromosome partitioning; E, Amino acid transport and metabolism; F, Nucleotide transport and metabolism; G, Carbohydrate transport and metabolism; H, Coenzyme transport and metabolism; I, Lipid transport and metabolism; J, Translation, ribosomal structure, and biogenesis; K, Transcription; L, Replication, recombination, and repair; M, Cell wall/membrane/envelope biogenesis; N, Cell motility; O, Posttranslational modification, protein turnover, chaperones; P, Inorganic ion transport and metabolism; Q, Secondary metabolites biosynthesis, transport, and catabolism; R, General function prediction only; S, Function unknown; T, Signal transduction mechanisms; U, Intracellular trafficking, secretion, and vesicular transport; V, Defense mechanisms; W, Extracellular structures; X, Unnamed protein; Y, Nuclear structure; Z, Cytoskeleton.

Because there is no transcriptomic data in the coumarin-producing plants, the transcriptomic dataset of *P. praeruptorum* was constructed and the results were summarized in Table S2 and Figure 3A. The dataset could also be retrieved from NCBI SRA sequence database under project accession number SRX997427 and the assemblies of the transcriptome constructed in this study are listed in Table S3. For short, 59,346,260 raw reads, 62,386 transcripts, and 40,952 unigenes were obtained. Based on the results of the *de novo* sequencing, assembly and annotation, the unigenes that hit in at least one database were discovered and subjected to functional classification (Figure 3). The results showed that the categories related to the biosynthesis and transport of metabolites, such as “metabolic process,” “catalytic activity,” “secondary metabolite biosynthesis,” and “transport” appeared in the three selected annotation and classification databases (Figures 3B–D). Thus, we found nearly all of the known genes encoding enzymes involved in the biosynthesis of the coumarin core compounds (Table 1). This result indicated that the method provided comprehensive and useful information on the function of the transcripts. Although the genes were annotated, the specific functions need to be verified. In addition, plenty of genes that did not participate in the target pathway need to be eliminated. Hence, a descent and specific dataset need to be constructed for further analysis.

**Table 1:** **The genes involved in coumarins biosynthesis**.

**Gene name**	**Enzyme No**.	**Numbers**
PAL	4.3.1.24	3
C4H	1.14.13.11	1
4CL	6.2.1.12	17
HCT	2.3.1.133	3
C3'H	1.14.13.36	1
CCoAOMT	2.1.1.104	3
CAOMT	2.1.1.68	7
CA2H		0
F6'H		2
C2'H		0
PS		5
AS		3
BMT	2.1.1.69	1
XMT	2.1.1.70	0

### Targeted metabolic pathways analysis to select candidate CYP450 and MDR genes involved in the biosynthesis and transport of coumarins

The main question resolved in this study was investigating the genes involved in coumarins biosynthesis and transport. Hence, the genes that may participate in this process were selected to narrow down the dataset. As shown in Figure 1, the enzymes involved in the monooxygenase reaction and hydroxylation reaction are largely unknown although some reports related to the reaction of core compounds formation had been published (Karamat et al., 2012, 2014; Vialart et al., 2012). Hence, the genes involved in monooxygenase reaction and hydroxylation reaction were selected as candidate genes. For example, cinnamate 4-hydroxylase that converts cinnamic acid to 4-coumaric acid is a CYP450 monooxygenase in the CYP73A family (Teutsch et al., 1993) and psoralen synthase that converts (+)-marmesin to psoralen is also a monooxygenase in the CYP71AJ family (Larbat et al., 2007, 2009). Therefore, the genes annotated as CYP450 genes, particular those in the CYP71AJ, CYP73, CYP84, and CYP98 clans which had been reported to be involved in coumarins biosynthesis were chosen for investigation (Bourgaud et al., 2006; Mizutani and Ohta, 2010; Nelson and Werck-Reichhart, 2011) (Table 2, Table S4). Hence, it could narrow down the query coverage (Bourgaud et al., 2006).

**Table 2:** **Summary of the CYP families and ABC families in the *P. praeruptorum* transcriptome dataset**.

**Clans**	**Gene**	**Gene No**.	**Clans**	**Gene**	**Gene No**.
CYP71	A1,A2,A6,A8,A9,A22,		CYP704	C1	4
	A23,A25,A26,AJ1,AJ2,		CYP707	A1,A3,A4,A7	9
	AJ3,AJ4,B10,B12,B14,		CYP710	A1 A1 B1,B2 B1 A1,A2 A1,A4 A1	2
	B23,B34,B36,B37,BL3,		CYP711	A1	1
	D6,D7,D8,D9,D11,D12,		CYP714	A1	2
	D55,E1	61	CYP716	B1,B2	7
CYP72	A1,C1	12	CYP724	B1	3
CYP73	A10	1	CYP725	A1,A2	2
CYP74	A	1	CYP734	A1,A4	3
CYP75	A2,B1,B2	4	CYP735	A1	2
CYP76	A1,A2,B1,B6,B10, C1,		Others		8
	C2,C3,C4	25	Total		239
CYP77	A1,A3,A4	5			
CYP78	A5,A6,A9	3	ABCA	1,2,4,7,8	9
CYP80	B2	1	ABCB	1,2,4,6,9,10,11,13,15,	47
CYP81	D1,E1,F1	14		19,20,21,25,26,28,29	
CYP82	A1,A3,A4,C2,C4	13	ABCC	1,2,3,5,8,9,10,12,13,	
CYP83	A1,B1	2		14,15	55
CYP84	A1	6	ABCE	2	3
CYP85	A1	5	ABCF	1,3,4,5	9
CYP86	A1,A2,B1	7	ABCG	1,3,4,5,7,8,10,11,12,14,	
CYP89	A2,A9	4		15,16,20,21,22,25,27,	
CYP90	A1,B1,D1,D2	5		28,29,32,35,40,41,43	72
CYP93	A2,A3	4	ABCI	1,6,8,10,11,19,20	8
CYP94	A1,A2	12	ABCPDR	1,2,3,4,7,12,15	28
CYP97	A3,B2,C1	8	Others total		5
CYP98	A2,A3	3	Total		236

As an important complement to secondary metabolites biosynthesis, the transporters involved in the transport of plant secondary metabolites play an increasingly significant role in biosynthetic studies (Yu and De Luca, 2013). Firstly, the differential subcellular localizations of the enzymes involved in biosynthesis indicate that compounds are synthesized in different organelles or tissues (Yazaki, 2005, 2006; Rea, 2007; Zhao and Dixon, 2010). Secondly, some of the genes responsible for the formation of plant secondary metabolites may be highly expressed in one tissue, whereas the metabolites may mainly accumulate in another tissue or organ (Yazaki, 2005, 2006; Rea, 2007; Zhao and Dixon, 2010). Hence, the pathway intermediates must be transferred between these locations and tissues to allow the enzymes, which displayed different subcellular localizations, to participate in the synthetic pathways (Yazaki, 2005). This just happened in coumarin compounds which were synthesized via phenylalanine in different organelles and then accumulated in different tissues and/or organelles for defense against pathogenic infections or other purposes. However, unlike the case for the genes that participate in coumarins biosynthesis, the transporters involved in coumarins transport have not been identified to date (Karamat et al., 2012, 2014; Vialart et al., 2012). And, no genes were annotated as being associated with coumarins transport despite of a large number of genes hit, annotation and classification as transporters (Figure 3). Of all the transcripts annotated for transport, the ATP-binding cassette (ABC) transporter, particularly the MDR (multidrug resistance protein, ABCC) have been reported to be associated with the transport of metabolites or to participate in transmembrane transport and/or the regulation of other transporters (Yazaki, 2005; Rea, 2007). Hence, the transcripts annotated as MDR were selected for investigating the genes involved in the transport of coumarins. In conclusion, 19 of 239 CYP450 genes and 55 of 236 ABC transporter genes were selected as candidate genes involved in the biosynthesis and transport of coumarin compounds according to previous studies (Yazaki, 2005; Mizutani and Ohta, 2010; Nelson and Werck-Reichhart, 2011) (Table 2, Table S5).

### MeJA-induced gene expression analysis to narrow down the candidate CYP450 and MDR genes

**Figure 4:**
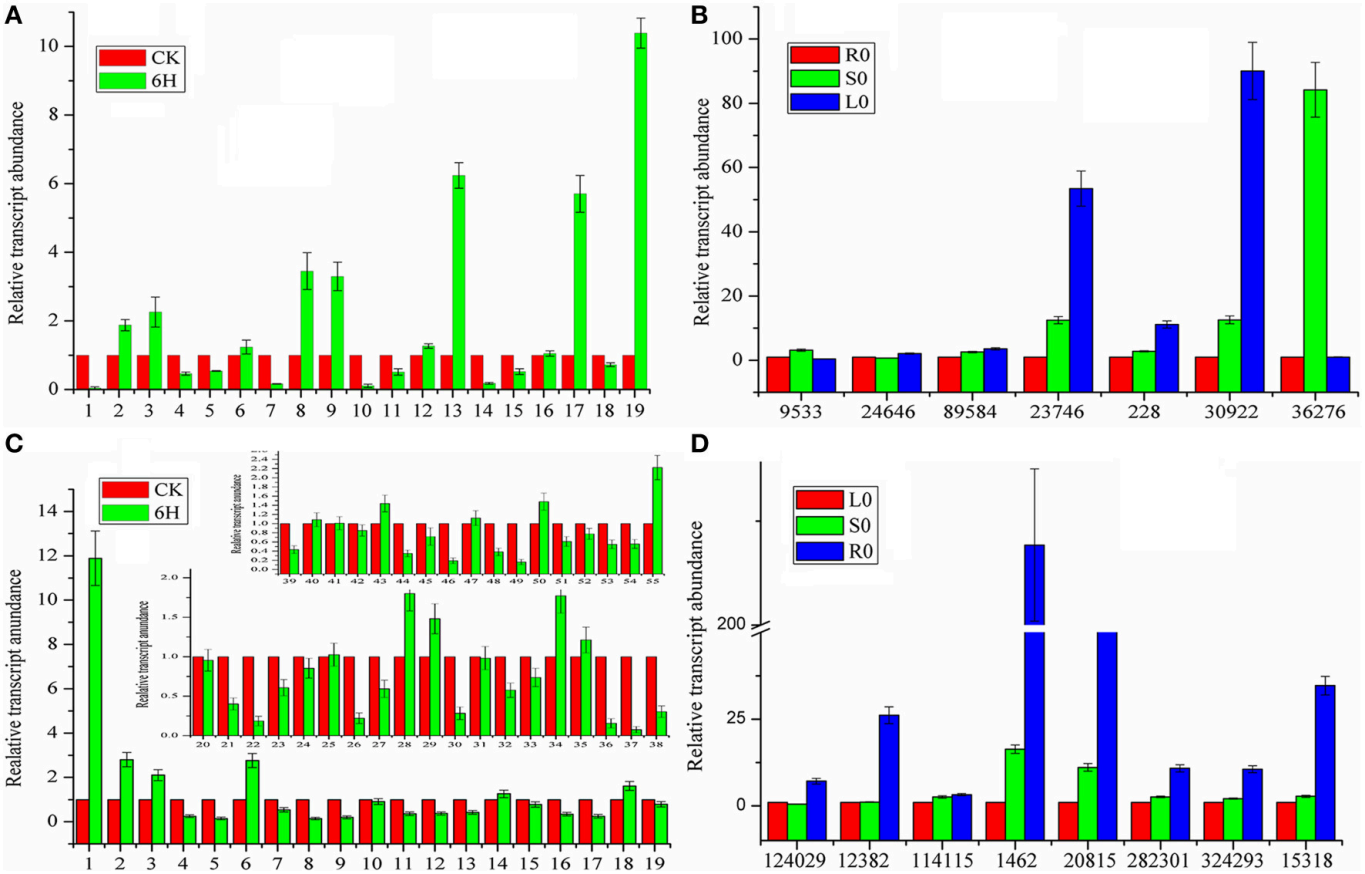
**Expression profiles of CYP450 and MDR genes involved in coumarins biosynthesis and transport. (A)** CYP450 genes expression after MeJA treatment. **(B)** Tissue-specific expression of CYP450 genes. **(C)** MDR genes expression after MeJA treatment. **(D)** Tissue-specific expression of MDR genes. CK represents the materials without treatment; 6H represents the materials treated for 6 h with MeJA. R0, S0, L0 represents the untreated materials of roots, stems, and leaves, respectively. For relative quantification, the gene expression levels of R0 and L0 were set as reference in each group of **(B,D)**. Each bar represents the mean value results from the mean of triplicate experiments ± standard deviation (SD).

**Figure 5:**
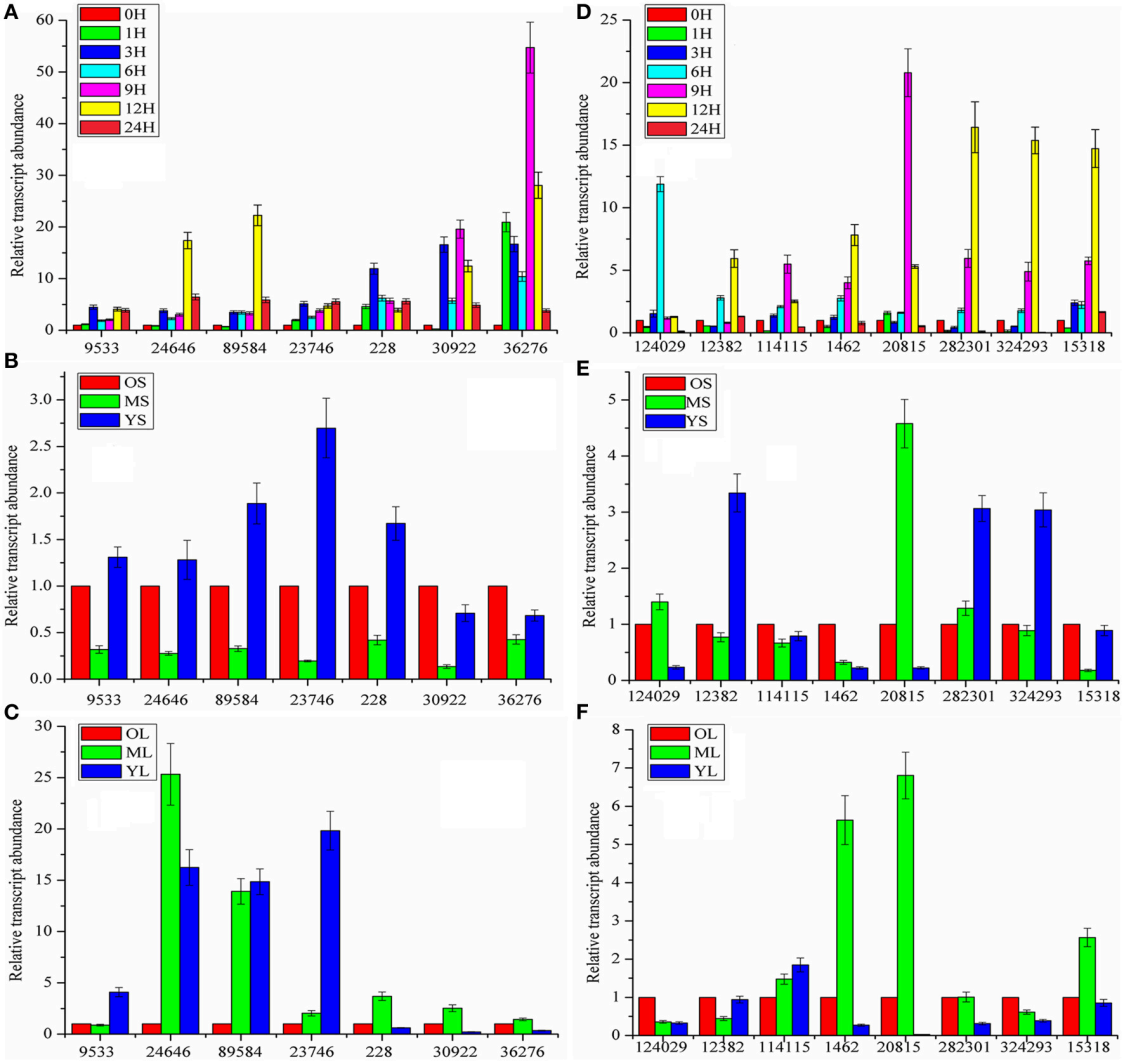
**Time and growth-stage-specific gene expression. (A,D)** Time-dependent expression levels of CYP450 and MDR genes. **(B,E)** Expression levels of CYP450 and MDR genes in stems (S) at different growth stages. **(C,F)** Expression levels of CYP450 and MDR in leaves (L) at different growth stages. 0, 1, 3, 6, 9, 12, 24H represent the time interval after MeJA addition; O, M, Y: short for old, mature, and young, respectively. For relative quantitative, the gene expression levels of 0H **(A,D)**, OS **(B,E),** and OL **(C,F)** was set as reference in each group. Each bar are presented as mean of triplicate experiments±standard deviation (SD).

Although a large number of genes that did not participate in the biosynthesis or transport of coumarin compounds were eliminated, it was not practical to simultaneously analyze all of the genes above. In addition, not all genes interested us. Therefore, MeJA-induced gene expression analysis was conducted to further screen the genes actually involved in the biosynthesis and transport of coumarin compounds based on the fact that the MeJA signaling pathway is generally regarded as a transducer of elicitor-signal transduction that leads to the biosynthesis of plant secondary compounds (Zhao et al., 2005; Mizutani and Ohta, 2010; Nelson and Werck-Reichhart, 2011). For example, the exogenous application of MeJA resulted in elevated levels of active enzymes in soybean cell-suspension cultures and the accumulation of high amounts of phenolics and lignins (Lois et al., 1989; Mandal, 2010). Hence, members of the CYP71AJ, CYP73, CYP84, and CYP98 clans were selected as candidate genes and then subject to qRT-PCR-based expression analysis. As shown in Figure 4A, the genes labeled as 2, 3, 8, 9, 13, 17, and 19 (corresponding to *comp228_c0_seq1, comp30922_c1_seq1, comp36276_c0_seq5, comp9533_c0_seq1, comp24646_c0_seq1, comp89584_c0_seq1*, and *comp23746_c0_seq1* in Table S1 and abbreviated to *228, 30,922, 36,276, 9533, 24,646, 89,584*, and *23,746*, respectively) displayed up-regulated expression. Then, the up-regulated genes were further subject to tissue-specific expression analysis (Figure 4B) and time-dependent induction experiment (Figure 5A). It indicated that *30,922* expressed at the highest level in the leaves, whereas *36,276* expressed at the highest level in the stems, and there was no difference in expression levels of 89,584 in three tissues. The time-dependent induction experiment showed that all of the genes displayed different modes of expression. For instance, MeJA had little effect on the expression level of *9533* but had a strong effect on *36276*. *228* responded immediately after the addition of MeJA, whereas *30,922* did not respond for 3 h (Figure 5A). The trend in the responses showed an up-down regulated expression pattern that was consistent with the results of the previous study (Belhadj et al., 2008). It has been reported that the genes in tissues of different states (young or old) displayed a different expression pattern and this pattern was related to the accumulation of compounds (Li et al., 2002; Sun et al., 2011). Thus, the expression levels of the selected genes in tissues with different physiological statuses were analyzed (Figures 5B,C). The results showed that high levels of expression occurred in young stems, whereas low levels of expression occurred in mature tissue. However, the results for the leaves were partially opposite of those for the stems, that is, *228, 30,922, 36,276*, and *24,646* were expressed at a higher level in mature leaves than in young leaves. Interestingly, old leaves tend to have low expression levels due to their physiological status of no longer participating in the biosynthesis of metabolites (Li et al., 2002).

**Figure 6:**
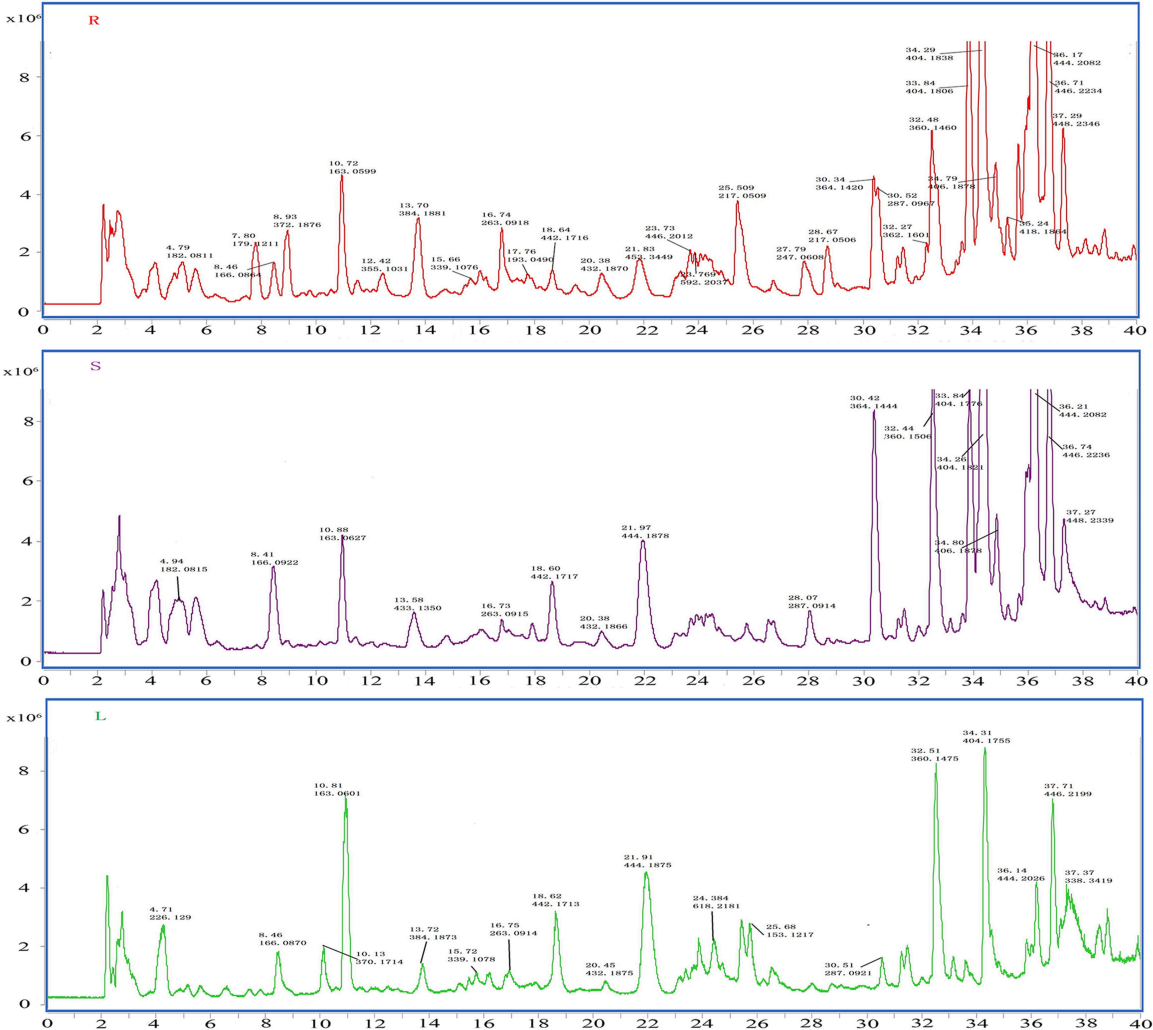
**MS TIC chromatograms of roots (R), stems (S), and leaves (L) of *P. praeruptorum* by HPLC/Q-TOF MS in positive ion mode**. Peak is marked by retention time and measured molecular weight.

Similar to the procedure above, 55 MDR transporters were first selected from the 236 ABC-transporter transcripts and then, the corresponding genes were screened using MeJA-induced expression analysis. As shown in Figure 4C, 1, 2, 3, 6, 28, 29, 34, and 55 (corresponding to *124,029, 114,115, 12,382, 1462, 20,815, 282,301, 324,293*, and *15,318*) displayed up-regulated expression. The tissue-specific expression analysis showed that all of the genes highly expressed in roots. Interestingly, *1462* and *20,815* had the highest expression levels and their expression levels in stems were also higher than those of the other MDR-transporter genes, whereas, the others showed no difference in the expression levels of the stems and leaves (Figure 4D). The time-dependent MeJA-induced expression analysis showed that these genes had the same expression pattern with CYP450s (Figure 5D). Analysis of the expression profiles of the selected genes at different physiological statuses revealed that they expressed at a higher level in mature leaves and had a lower expression level in young leaves (Figure 5F). However, this trend was partially reversed in the stems and a relatively higher expression level was found in young stems (Figure 5E). The differential expression behavior was related to the profiles of the secondary metabolites in different tissues.

Above all, the up-/down-regulated expression patterns were consistent with those observed in the previous study (Belhadj et al., 2008) (Figures 5A,D). Upon MeJA treatment, only 7 of 19 CYP450 genes and 8 of 55 MDR transporters displayed an increased expression level, which greatly reduced the number of genes to be evaluated. While, whether all of the genes we investigated participated in the biosynthesis and/or transport of coumarin compounds and whether these genes were associated with the compounds accumulation depended on the quantitative analysis of coumarin contents.

### Metabolite analysis and structural identification

To identify the coumarin compounds in *P. praeruptorum*, HPLC coupled with Q-TOF MS was used because this method was rapid and could provide accurate mass measurements (Ling et al., 2013). Additionally, the peak areas could also be used for relative quantification (Zhang et al., 2014). However, in our first attempt to utilize this method, less than 10 peaks were obtained using HPLC. The same problem occurred in LC-MS (data no given). Considering that the peaks corresponding to the high-abundance compounds (such as praeruptorin A and praeruptorin C) may hide those peaks corresponding to the low-abundance compounds, a C18E cartridge was used to separate the two fractions for the first time according to the method described in materials and methods part. After this process of treatment, peaks of low-abundance compounds were observed in the extracts using HPLC and LC-MS (Figure 6), which made it possible to analyze compound contents. Figure 6 shows the distinct differences in the contents of the compounds and their peak numbers, and it can be easily observed that many low-abundance compounds were identified in the extracts of the roots, stems, and leaves. Interestingly, roots tend to have more numbers of compounds and higher peak intensity than stems and leaves which could account for why people choose roots as medical parts (Commission, 2010). Another phenomenon is that, in a “relative” point of view, the leaves seem have higher ratio of maximal polar components than stems and roots. This could be interpreted as that leaves are the main sits for biosynthesis, hence, the maximal polar components (such as umbelliferone) mainly existed in this tissue (Figure 6). Table 3 shows the 19 coumarin compounds (the structures are listed in Figure S1) identified by their signals in the corresponding extracted-ion chromatograms and their second-order fragment ion. Their identities were confirmed by previous studies or standard compounds. Here, we took (±)-Praeruptorin A (Pd-Ia) as an example to illustrate the process of identification for it constituted the main composition of *P. praeruptorum* (Kong et al., 1996). Firstly, according to the mass spectrometry data, the exact mass of the quasi-molecular ion 404.1704 (m/z, [M+NH_4_]^+^) and the MS/MS fragment ions 245.0808, 227.0699, 175.0385, and 83.0502 (m/z) were obtained (Figure S2). And then, Agilent LC-Q-TOF-MS MassHunter Qualitative Analysis Software was used to calculate the elemental composition and five different possible element compositions were produced. Thirdly, the MS, MS/MS fragments and elemental composition all compared to available literature information, combined with the deduced fragmentation pathway (Figure S3), (+)-Praeruptorin A (Pd-Ia, C_24_H_26_O_7_) was found to be the most possible compound. For further confirmation, standard compound was used and the result was consistent with the identification outcome. Subsequently, another 18 coumarin compounds were identified.

**Table 3:** **Compounds detected in the extracts of *P. praeruptorum* by HPLC-Q-TOF-MS/MS**.

**Formula**	**Identification**	**t_R(min)_**	**Monitoring ion**	**Measured (m/z)**	**Calculated (m/z)**	**Error (ppm)**	**Major fragment ions (m/z)**
C_9_H_6_O_3_	Umbelliferone	10.724	[M+H]^+^	163.5099	163.1421	0.17	135.0442,119.0493
C_16_H_18_O_9_	Scopolin	12.423	[M+H]^+^	355.1029	355.1024	−1.62	163.0385,145.0284,135.0440,117.0335
C_16_H_18_O_8_	4-Methylumbelliferyl glucoside	15.669	[M+H]^+^	339.1073	339.1072	0.47	147.0438,119.0490,91.0548
C_14_H_14_O_5_	Khellactone	16.749	[M+NH_4_]^+^	263.0918	263.0914	−1.68	229.0862,203.0700,175.0391,59.0506
C_10_H_8_O_4_	Scopoletin	17.662	[M+H]^+^	193.0493	193.0495	1.43	178.0255,150.0308,133.0281,122.0360,94.0417
C_20_H_24_O_10_	Apterin	18.641	[M+NH_4_]^+^	442.171	442.1708	−0.45	245.0799,203.0694,187.0381
C_20_H_26_O_10_	Praeroside VI	21.974	[M+NH_4_]^+^	444.1864	444.1871	−1.63	247.0962,229.0858,175.0388
C_28_H_30_O_13_	Praeroside I	23.761	[M+NH_4_]^+^	592.2028	592.2025	−0.58	313.0894,263.0912,151.0377
C_12_H_8_O_4_	5-Methoxypsoralen	25.501	[M+H]^+^	217.0492	217.0495	1.29	202.0252,174.0306,146.0275,118.0411,90.0466
C_12_H_8_O_4_	8-Methoxypsoralen	28.677	[M+H]^+^	217.0501	217.0495	−2.75	202.0256,174.0360,146.0385,118.0412,90.0468,77.0396
C_18_H_18_O_7_	Qianhucoumarin D	30.344	[M+NH_4_]^+^	364.14	364.1391	−2.61	245.0810,227.0703,175.0389
C_19_H_20_O_6_	Qianhucoumarin A	32.271	[M+NH_4_]^+^	362.1606	362.1598	−2.21	243.0644,213.0694,57.0708
C_19_H_18_O_6_	Pd-Ib	32.463	[M+NH_4_]^+^	360.1447	360.1442	−1.49	243.0655,215.0704,83.0501,55.0556
C_21_H_22_O_7_	(±)-Praeruptorin A(Pd-Ia)	34.296	[M+NH_4_]^+^	404.1705	404.1704	−0.29	245.0808,227.0699,175.0385
C_21_H_24_O_7_	Peucedanocoumarin I	34.792	[M+NH_4_]^+^	406.1863	406.186	−0.68	245.0806,227.0698,175.0383,85.0651,57.0708
C_22_H_24_O_7_	Qianhucoumarin J	35.243	[M+NH_4_]^+^	418.1856	418.186	0.97	227.0699,83.0498,55.0552
C_24_H_26_O_7_	(+)-Praeruptorin B(Pd-II)	36.172	[M+NH_4_]^+^	444.2104	444.2017	0.6	327.1219,227.0688,83.0498,55.0553
C_24_H_28_O_7_	Praeruptorin F	36.713	[M+NH_4_]^+^	446.2176	446.2173	−0.57	245.0801,227.0696,175.0384,83.0499,55.0553
C_24_H_30_O_7_	Praeruptorin G	37.293	[M+NH_4_]^+^	448.2334	448.233	−1.04	245.0800,227.0695,175.0381,85.0659,57.0708

### Metabolomics analysis and potential biomarkers exploration

**Figure 7:**
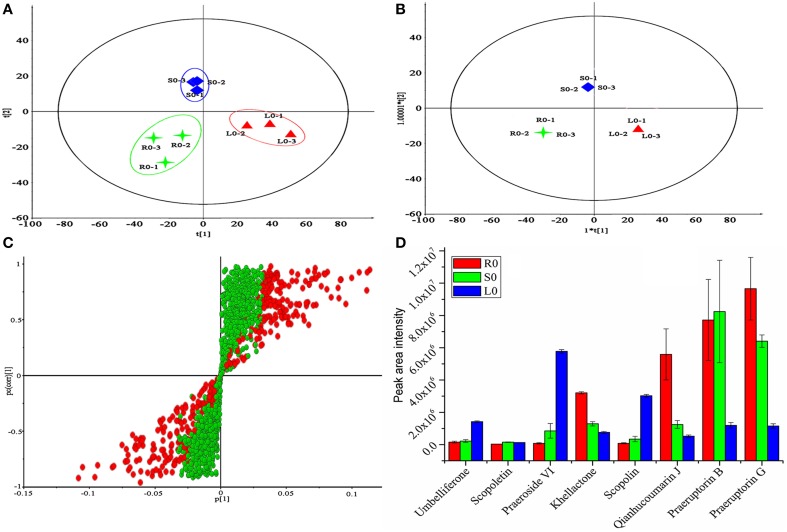
**Metabolomics analysis of *P. praeruptorum* extracts. (A)** PCA scores plots. **(B)** OPLS-DA score plots. **(C)** OPLS-DA loadings plot derived from HPLC-Q-TOF-MS data of roots (R0), stems (S0), and leaves (L0). **(D)** Peak intensity of eight compounds between the three tissue groups. Each bar represents the mean value results from triplicate experiments ± standard deviation (SD); 1, 2, 3 represent the three parallel tests and 0 represent the tissue used in this study was no treated with MeJA.

After the method of separation was established, discovering the significant difference compounds for a correlation study of gene expression and metabolites accumulation was urgent. To solve this problem, metabolomics analysis was conducted due to its powerful performance in natural product discovery (Yan et al., 2015). At first, HPLC combined with Q-TOF MS was first conducted for data collection, and then the data were normalized according to the method described below in materials and methods part. Subsequently, the 1713 normalized variables were imported into the SIMCA-P V13.0 (Umetrics, Sweden) platform for multivariate analysis. The PCA score plots (Figure 7A) showed that the components of the roots, stems and leaves were clearly clustered into three groups. The OPLS-DA scores also showed that the components of the roots, stems and leaves fell into well-distinguished classes (Figure 7B, R^2^X = 0.957, Q^2^ = 0.885, were acceptable), indicating that the study had good levels of predictability and reliability. OPLS-DA loading plots were used to determine which compounds contributed highly to the differences among the three tissues. As shown in Figure 7C, 405 of 1713 variables with a VIP value of >1.000 were denoted as potential contributors and they were colored in red. To select potential biomarkers for a correlation study, student's *t*-test was used with the *P*-value set to 0.05 to designate significantly different variables. After two rounds of selection, 65 marker metabolites were selected. However, not all of the 65 marker metabolites were coumarin compounds which we were mainly interested in. Hence, the metabolites identified in Table 3 were used as a coumarin compounds reference dataset for further selection. Finally, 8 significantly different coumarin compounds were identified as potential biomarkers for further study. For example, the peak intensities of coumarin compounds present at different levels in the roots, stems and leaves were first investigated, and the results are shown in Figure 7D. The result indicated that there was an obvious difference in the contents of the roots, stems, and leaves. These differences were consistent with the results listed in Figures 4, 6 in some extent and it may be related to the levels of expression of the genes involved in their biosynthesis and transport.

### Merging the gene expression and compound accumulation to identify the genes involved in coumarins biosynthesis and transport

After the candidate genes and potential biomarkers were discovered, the relationship between gene expression and compound accumulation arose. To address these issues, the differences in the peaks intensity of the coumarin compounds in roots, stems and leaves were investigated, and the results are shown in Figure 7D. As shown, the levels of umbelliferone, praeroside VI and scopolin were higher in leaves, whereas those of khellactone, qianhucoumarin J, praeruptorin B, and praeruptorin G were higher in roots and stems. The reason for these phenomena was that the former compounds synthesized during the core formation and the latter required prenylation, hydroxylation or cyclization or other structural modifications (Figure 1). Consistent with the tissue-specific difference in the level of compounds accumulation, the related gene expression level also displayed a tissue-specific pattern (Figure 4B). In particular, the expression level of *23,746, 228*, and *30,922* tended toward a leaves-specific expression pattern and those of *36,276* and *9533* tended toward a stems-specific expression pattern. These results indicated that *23,746, 228*, and *30,922* may be related to the formation of coumarin core compounds and *36,276* and *9533* participated in the prenylation, hydroxylation and cyclization or structural modification. Considering the tissue-specific patterns of gene expression, the question is how these processes occur. As is known to all, different compounds were synthetized not only in different tissues but also in different organelles, which was proved by subcellular localization experiments (Karamat et al., 2014). For instance, CYP450 proteins are heme-thiolate membrane-bound proteins that are generally bound to the surface of the endoplasmic reticulum (Kawai et al., 2014), whereas O-methyltransferase tended to express throughout the cytoplasm (Yazaki, 2005) (our unpublished data). Moreover, the leaves and stems are likely to be the main sites of coumarins biosynthesis, but these compounds are localized mainly in the roots which are the medicinal portions of *P. praeruptorum* (Commission, 2010). Given the existence of transporters, these facts appear to be understandable, and there are many reports concerning the transporters (Li et al., 2002; Yazaki, 2005; Rea, 2007; Yu and De Luca, 2013). As shown in Figure 4D, the selected transporters were expressed at a higher level in the roots than in other tissues, which was consistent with the differential content levels of the relevant compounds (Figure 7D). This phenomenon indicated that the selected genes participated in the transport of coumarin compounds. Does a transporter translocate a specific compound or perhaps two/more compounds? It was widely accepted that ABC transporters have broad substrate specificity (Yazaki, 2006). However, the results of our study indicated that a transporter specifically translocated one kind of compounds. Although all of the selected genes were highly expressed in the roots, *1462, 20,815*, and *15,318* tended to highly express in mature or young leaves and *124,029* and *324,293* tended to highly express in old leaves (Figures 5E,F). The results indicated that the former transcripts may participate in the transport of coumarins core compounds and the latter transcripts may participate in the transport of the completed coumarin compounds. This tendency for a certain compound to be transported via a various mechanism was analyzed using vacuolar-membrane vesicles purified from the red beets of *Beta vulgaris*. The results demonstrated that two phenol glucosides, p-hydroxycinnamic acid, and p-hydroxybenzoic acid, were apparently transported via a H^+^-gradient-dependent mechanism, the glutathione conjugate of an analog of the herbicide chlorsulfuron appeared to be transported via an ABC transporter (Yazaki, 2006). Transport mechanisms may also function in *P. praeruptorum*, and this is why its roots are generally chosen for medicinal usage (Commission, 2010). As shown in Figure S4, the content of “made-up compound,” such as qianhucoumarin J and praeruptorin G, accumulated in a time-dependent pattern but the content of the coumarin core compounds (such as umbelliferone and scopoletin) did not change over time. The results also showed a decreasing content of the 8 selected compounds. This phenomenon was consistent with the fact that this type of plant is generally being harvested at 1 or 2 year after planting, and its quality would affect being grown for a long period particularly after it had bloomed.

## Conclusions

In this study, a large amount of transcriptomic data for *P. praeruptorum* was assembled for the first time. After sequence assembly and functional annotation, the putative CYP450 and MDR genes involved in the biosynthesis and transport of coumarin compounds were selected. Based on the results of the MeJA-induced expression analysis, 7 CYP450s and 8 MDR transporter unigenes were selected as candidate transcripts. Then, HPLC-Q-TOF-MS/MS-based metabolomics analysis were merged to identify the differential chemical constituents and quantify their contents in roots, stems and leaves for a correlation study of gene expression and metabolites accumulation in coumarins biosynthesis and transport. The results indicated that *23,746, 228*, and *30,922* may be related to the formation of the coumarin core compounds whereas *36,276* and *9533* participated in the prenylation, hydroxylation, cyclization or structural modification. It appeared that *1462, 20,815*, and *15,318* participated in the transport of coumarin core compounds while *124,029* and *324,293* participated in the transport of the “made-up cumpounds.” Additionally, studies of the tissue-specific, growth-stage-specific, and time-dependent accumulation of coumarin compounds were performed for the first time. The results would facilitate investigations of the biosynthesis and transport of coumarin compounds and promote the medicinal usage of components of *P. praeruptorum*. In addition, the method used in this study would also provide a guideline to gene discovery of other non-model plants.

## Author contributions

LK and YZ conceived and designed the experiments and contributed the reagents/materials/analysis tools. QZ and SX analyzed the data and wrote the manuscript. CH, JX, and MC performed the RNA isolation experiment. JL and TL provided the *P. praeruptorum* materials and participated in the design of the study. CH, SX, and JX helped to analyze the data and draft the manuscript. YC coordinated the study and revised the manuscript. SX, CH, JX, and MC participated in picture editing. LK, CY, JL, TL, and QZ conducted in compound identification. All authors read and approved the final manuscript.

### Conflict of interest statement

The authors declare that the research was conducted in the absence of any commercial or financial relationships that could be construed as a potential conflict of interest.
