# Supplementary material for: Integration of a Decrescent Transcriptome and Metabolomics Dataset of Peucedanum praeruptorum to Investigate the CYP450 and MDR Genes Involved in Coumarins Biosynthesis and Transport
Source: Front Plant Sci. 2015 Dec 10;6:996. doi: 10.3389/fpls.2015.00996 (PMC4674560; doi:10.3389/fpls.2015.00996)
Supplement: Supplementary file 1 [file DataSheet1.DOCX]

***Supplementary Material***

**Integration of a** [**decrescent**](javascript:showjdsw('showjd_0','j_0')) **transcriptome and metabolomics dataset of *Peucedanum praeruptorum* to investigate the CYP450 and MDR genes involved in coumarins biosynthesis and transport**

Yucheng Zhao^1^, Tingting Liu^1^, Jun Luo^1^, Qian Zhang^1^, Sheng Xu^2^, Chao Han^1^, Jinfang Xu^1^, Menghan Chen^1^, Yijun Chen^1^ and Lingyi Kong^1^*

***Correspondence:**

Lingyi Kong

[cpu_lykong@126.com](mailto:cpu_lykong@126.com)

**Lengends of supplementary materials**

Table S1 Primers used in this study

Table S2 Statistics results of transcriptome data

Table S3 Assemblies of the transcriptome constructed in this study.

Table S4 Putative CYP450 genes in the *P. praeruptorum* cDNA library

Table S5 Putative ABC genes in the *P. praeruptorum* cDNA library

Figure S1 The structure of compounds were identified from the extracts of *P. praeruptorum* by HPLC-Q-TOF-MS/MS.

Figure S2 Primary scan **(A)** and secondary fragments **(B)** of (+)-praeruptorin A.

Figure S3 The deduced [fragmentation pathway](http://dict.cnki.net/dict_result.aspx?searchword=%e8%b4%a8%e8%b0%b1%e8%a3%82%e8%a7%a3%e8%a7%84%e5%be%8b&tjType=sentence&style=&t=fragmentation+regularities) of (+)-praeruptorin A.

Figure S4 Contents variation of marker compounds.

Y1, Y2, Y3 represent one, two and three year after planting, respectively. For relative [quantification](http://dict.youdao.com/w/quantification/), the content of Y1 was set as reference in each group, each bar are presented as mean of triplicate experiments±standard deviation (SD).

## Supplementary Tables

| Table S1 Primers used in this study | | | | |  |
| --- | --- | --- | --- | --- | --- |
| Primers of putative CYP450s for expression analysis | | | | |  |
| Serial No. | Gene_ID | | F (5'to3') | | R (5'to3') |
| 1 | comp35081_c0_seq10 | | AAGGTCGTCGCTTGAAGCC | | GTTCCATGGCTCTCCTGGG |
| 2 | comp9533_c0_seq1 | | CCCATAATGTCCCTTCGCC | | CCAGCTGTACTGTGGGACG |
| 3 | comp24646_c0_seq1 | | CGGGATTGAAGGGAAAGGT | | GCCAGCATCTGTACCCTGA |
| 4 | comp30862_c1_seq2 | | TATTTCCACCTTTCCCTGC | | ATTCCACTGCTCTCCTGGG |
| 5 | comp30862_c0_seq1 | | AGGGTTAATTCCTTTCGCAG | | GTGCAACCCGGCATAGTACA |
| 6 | comp12917_c0_seq1 | | TGTTTCGAAAAATCTGCCACCT | | TAATGGACCATACTTTTGAGCC |
| 7 | comp9524_c0_seq1 | | TCAACCAGCAAAAATCTGCCA | | TCTTAGTGCTATGTGCGGGTC |
| 8 | comp89584_c0_seq1 | | AGAAATAGGCAAGGGCAAATC | | GTTCACATCTTGCCTTGCTAC |
| 9 | comp23746_c0_seq1 | | AAGATGTGATCAATGGCGC | | TTTATCCCTGTTCTTCGCC |
| 10 | comp1195_c0_seq1 | | CCATTTGGCTCTGGAAGAA | | AGCCCGAATTCCTCAGTCA |
| 11 | comp11095_c0_seq1 | | TAACGTCCCGAAAGCTGCC | | TAACGTCCCGAAAGCTGCC |
| 12 | comp201804_c0_seq1 | | TATGGCCACAAGGCGAGTG | | TGGGTCAGGTCGAAGGTCC |
| 13 | comp228_c0_seq1 | | TCCCTCCAAACATTACGTCC | | GGTTCTATTGGCGAGGTGAA |
| 14 | comp193708_c0_seq1 | | GGCATGGACAAGATGGTGA | | TGGTGCAGTCCTCTATGCA |
| 15 | comp600723_c0_seq1 | | TGCCATGGCAACCATCTTC | | CCATGCGGAGATGGAAAAG |
| 16 | comp30922_c0_seq1 | | GCCTTGAACTCCTTGCCTT | | AGGCAGTATCTTGGAGGGG |
| 17 | comp30922_c1_seq1 | | TTCCTAATTGTGCCCCAGG | | CGTGACCCTGCTGTTTGGA |
| 18 | comp31295_c0_seq1 | | CAACAAACTCTTCCCCTGC | | GCTTTTTACTCCCAAGCGA |
| 19 | comp36276_c0_seq5 | | CTGGATATGTTCGCTGCTG | | TTGCCTTTGCCTATTTCTC |
|  |  | |  | |  |
| Primers of putative MDR for expression analysis | | | | |  |
| Number | | Gene_ID | | F (5'to3') | R (5'to3') |
| 1 | | comp124029_c0_seq1 | | CCAAATTGCAAAGGTTCCA | TCGGAACAATTGACCCAAG |
| 2 | | comp12382_c0_seq1 | | ATGGAATATTTGCAATGCCG | TAGTTCCGGTATCTGGCCAG |
| 3 | | comp114115_c0_seq1 | | TGGGACAAAGGCTTATGTTTCTC | CTTTGTTGGTCCATATTCTTCCC |
| 4 | | comp129698_c0_seq1 | | GTGCGAACTGGATGCTTTG | AAAGGCGTTCTGGATTTGG |
| 5 | | comp14341_c0_seq1 | | TTGAGGATAGTCGACCCCC | TTCCAATCTTCGTCCCACC |
| 6 | | comp1462_c0_seq1 | | AGCCCTACAAGTCCTGGAGC | TAATGCCACACTGGAGTGGC |
| 7 | | comp146802_c0_seq1 | | CAACCACTCAAGGCCATGG | GAGCGTCACCAGCAACACC |
| 8 | | comp14945_c0_seq2 | | GCTTCTAGCCAGGGTGCT | ACGGTTGGAATCCGATGA |
| 9 | | comp15366_c1_seq1 | | GTTATCCAGCATTTTGCGG | CTGTGAAACTTTGGCCGAG |
| 10 | | comp15366_c0_seq1 | | GCGCCCATGTCTTTCTTTG | CATGCCACTTGCGACATCA |
| 11 | | comp158090_c0_seq1 | | TACTTCCCAAGCCACCTGG | AAATCGGGCATCCACAGAC |
| 12 | | comp15961_c0_seq1 | | GGCCTTCTCTATTCGCAGC | AATCTCCGAGGGTTAGGGC |
| 13 | | comp16814_c0_seq1 | | GATCCAAGCTGGCTCCCTG | TCTGGTGGGGAGGCTTCTG |
| 14 | | comp172993_c0_seq1 | | AGAGCCATGACCCTTGCAG | CTCTGTTCAAGATGCGCCC |
| 15 | | comp179761_c0_seq1 | | TGCAGTGATTCGGCATCAG | GAGTTGGTTACCGCCTTCG |
| 16 | | comp192003_c0_seq1 | | AAAGCGCTGCTGCTAGGAG | GTGAAGTGAACGCCCTTGC |
| 17 | | comp192847_c0_seq1 | | CCGGAAGGAAGTCAACTTGG | TGATCCATTCAGCGCAGTTG |
| 18 | | comp20815_c0_seq1 | | GCAAGCTGGTACTGGTCCTC | CATCGTGCAATCCATCACTG |
| 19 | | comp21353_c0_seq1 | | TTCTAAAGGTGGGTTCGGC | TCCGTTATTCATGCTTCCG |
| 20 | | comp21919_c0_seq1 | | CTCTGCTTCTGTCCCCCAC | AAGCATGCTGCCTGAAACC |
| 21 | | comp235458_c0_seq1 | | TCTTTTGGGCCACAACACC | GGGAATGAGTTCAGCGGTG |
| 22 | | comp237808_c0_seq1 | | AGCCATGGCTGTTACGTGC | CCGCTCCAGAGACTGCAAG |
| 23 | | comp24452_c0_seq2 | | AATGCAATTCTTGGTCCCC | ACTGCGATGGGCTTCCTAC |
| 24 | | comp255732_c0_seq1 | | TCCAAGGTGTCTGCGCAAC | TGTGGTATGGTTGGCTCGG |
| 25 | | comp26956_c0_seq1 | | CGCTCCAGTTATCCCCTTC | AATTTGGACCCTCTAGGCG |
| 26 | | comp270361_c0_seq1 | | TGAATTGTTTCCCACTGGTG | CGCTAAAAGGGTCGTCAAG |
| 27 | | comp274132_c0-seq1 | | TAAGCGTTGATGGAGGCTG | TCTTTTCCGTGCTCCAATG |
| 28 | | comp282301_c0_seq1 | | GTGGCAATTGAAGCAAGCC | GACGGTGGATGCAGAGAGG |
| 29 | | comp2_c0_seq1 | | AATGTTTCCGAAGCAAGAGC | TGCCCGTGCACTATATCAAG |
| 30 | | comp31120_c0_seq2 | | TCCCCTCGAACATTGTTGG | TTTACAAGCCATCTGCCGG |
| 31 | | comp31372_c0_seq1 | | TGGTCCCAATCGATAGTGC | TGGATGGTGGTCAGCTCTC |
| 32 | | comp31833_c0_seq1 | | ACCGTAGGACAAAGCCATTC | ATTTTGCAGCAAATGAGTGG |
| 33 | | comp31833_c1_seq2 | | CGTCCAGCTGCACCACTTG | TTCCTCCTGAGGGCTCCAC |
| 34 | | comp324293_c0_seq1 | | TGCGGCCACTTTTCATCAC | TGCTTACTGGGGCTTTTGG |
| 35 | | comp32579_c0_seq2 | | AGTGGAAACCGTTGGCTGG | CTGGTTTTCTGCCCTTCCG |
| 36 | | comp33355_c1_seq10 | | TCCAAGCGAAAGGTCAGCC | CCAAGAATGCAGATTTCGGG |
| 37 | | comp34057_c0_seq1 | | CAACCGATGGGCAATAATG | CTTGATGAGGCGACAGCAG |
| 38 | | comp35587_c1_seq3 | | CGACAGTCCCGACAATTGC | GACAGGCAAGAAGGTTGCG |
| 39 | | comp35821_c0_seq4 | | ACAGGTTCGGTATGCTCCAC | TTCCCACTTCCTGTTCTTCC |
| 40 | | comp35905_c0_seq1 | | CCCCTCGTGAGGGAAAGTC | CGTTCTGGAAAGAGGCAGC |
| 41 | | comp35905_c1_seq8 | | AGCGTTACCACGAGGAAGC | ATGCCACAGACAGCGACAC |
| 42 | | comp36092_c0_seq4 | | TATTACGGTGCAGGTTGCG | TTAATTTGCTTGGGACGGG |
| 43 | | comp422878_c0_seq1 | | CCCACTCCCTGTTCTACCC | TTCATGTTCAGTACACCCCG |
| 44 | | comp869_c0_seq1 | | TGGTCGAACTGGAAGTGGG | GGAATAATCCCGAAACGGG |
| 45 | | comp161881_c0_seq1 | | AAGGACCAAGCTGAGGGAC | GGCCAGAGGTTGTGATACTG |
| 46 | | comp220134_c0_seq1 | | GAATCCCTGGTCCTGCTTC | TTATAGAAGGTTCGGGAGACC |
| 47 | | comp318012_c0_seq1 | | TCATGAAATAGCATGCCCAG | GGAAAGAAGAAGCAAGGTGG |
| 48 | | comp134033_c0_seq1 | | GGCTCTTGCATGGTGTTCC | CATCACAACATCCCCCACC |
| 49 | | comp14248_c0_seq1 | | CTATGGTAAAGGGTGGCGC | ACTGTGGGACAAGCCCATG |
| 50 | | comp222633_c0_seq1 | | ACATTTGCAGCCATCCAGG | AGTTGGAGATGCTGGCCTG |
| 51 | | comp11728_c0_seq1 | | AGTCCTCTTCCTTCTCAAAGC | TGGTCGAAAAAGTCAACCG |
| 52 | | comp268684_c0_seq1 | | CGAAAGCACCAACAGAGGC | ATGCCGAAACAATCCAAGG |
| 53 | | comp28447_c0_seq2 | | CAGTGTTCTGCGGTTGGTC | CCCAGTATGACAGCCACAGG |
| 54 | | comp33026_c0_seq11 | | AGAAAGCGCCAATCCTACC | GTGGCTTTCACTGCGTCTG |
| 55 | | comp15318_c0_seq1 | | TTTGTGTGCCTTGGTCGAA | AAAAGCAACAAAAGGCCGA |
|  | |  | |  |  |

Table S2 Statistics results of transcriptome data

| Table S2.1 Raw data information | |
| --- | --- |
|  | NGS2310NJ |
| Total Reads Count(#): | 59346260 |
| Total Bases Count(bp): | 5947619766 |
| Average Read Length(bp): | 100.2189484 |
| Q30 Bases Count(bp): | 3794352396 |
| Q30 Bases Ratio(%): | 63.79614947 |
| Q20 Bases Count(bp): | 4337379305 |
| Q20 Bases Ratio(%): | 72.92630457 |
| Q10 Bases Count(bp): | 4523466621 |
| Q10 Bases Ratio(%): | 76.05507411 |
| N Bases Count(bp): | 9366662 |
| N Bases Ratio(%): | 0.157485891 |

| Table S2.2 QC result | | | | | | | |
| --- | --- | --- | --- | --- | --- | --- | --- |
|  | Raw data | | | Clean data | | | |
| Sample | Raw sequences | Raw bases | Raw_mean_length | Good_sequences | Good ratio | Good bases | Good mean length |
| NGS2310NJ | 59346260 | 5947619766 | 100.2189484 | 46931496 | 79.08079801 | 4337830572 | 92.42898569 |

| Table S2.3 Assembly result | | | | | | | | | |
| --- | --- | --- | --- | --- | --- | --- | --- | --- | --- |
|  | All_num | >=500bp | >=1000bp | N50 | N90 | Max_len | Min_len | All_len | Mean_len |
| Transcript | 62386 | 33029 | 16502 | 1115 | 331 | 8556 | 201 | 47477509 | 761.03 |
| Unigene | 40952 | 18665 | 9543 | 1084 | 291 | 8556 | 201 | 28709076 | 701.04 |

| Table S2.4 GO annotation statistics | | |
| --- | --- | --- |
| Database | Number of Unigenes | Percentage(%) |
| Annotated in CDD | 15916 | 38.87 |
| Annotated in KOG | 12452 | 30.41 |
| Annotated in NR | 27885 | 68.09 |
| Annotated in NT | 10984 | 26.82 |
| Annotated in PFAM | 11512 | 28.11 |
| Annotated in Swissprot | 19971 | 48.77 |
| Annotated in TrEMBL | 27790 | 67.86 |
| Annotated in GO | 21467 | 52.42 |
| Annotated in KEGG | 4977 | 12.15 |
| Annotated in at least one database | 28228 | 68.93 |
| Annotated in all database | 2403 | 5.87 |
| Total unigenes | 40952 | 100 |

## Supplementary Figures





Figure S1 The structure of compounds were identified from the extracts of *P. praeruptorum* by HPLC-Q-TOF-MS/MS.


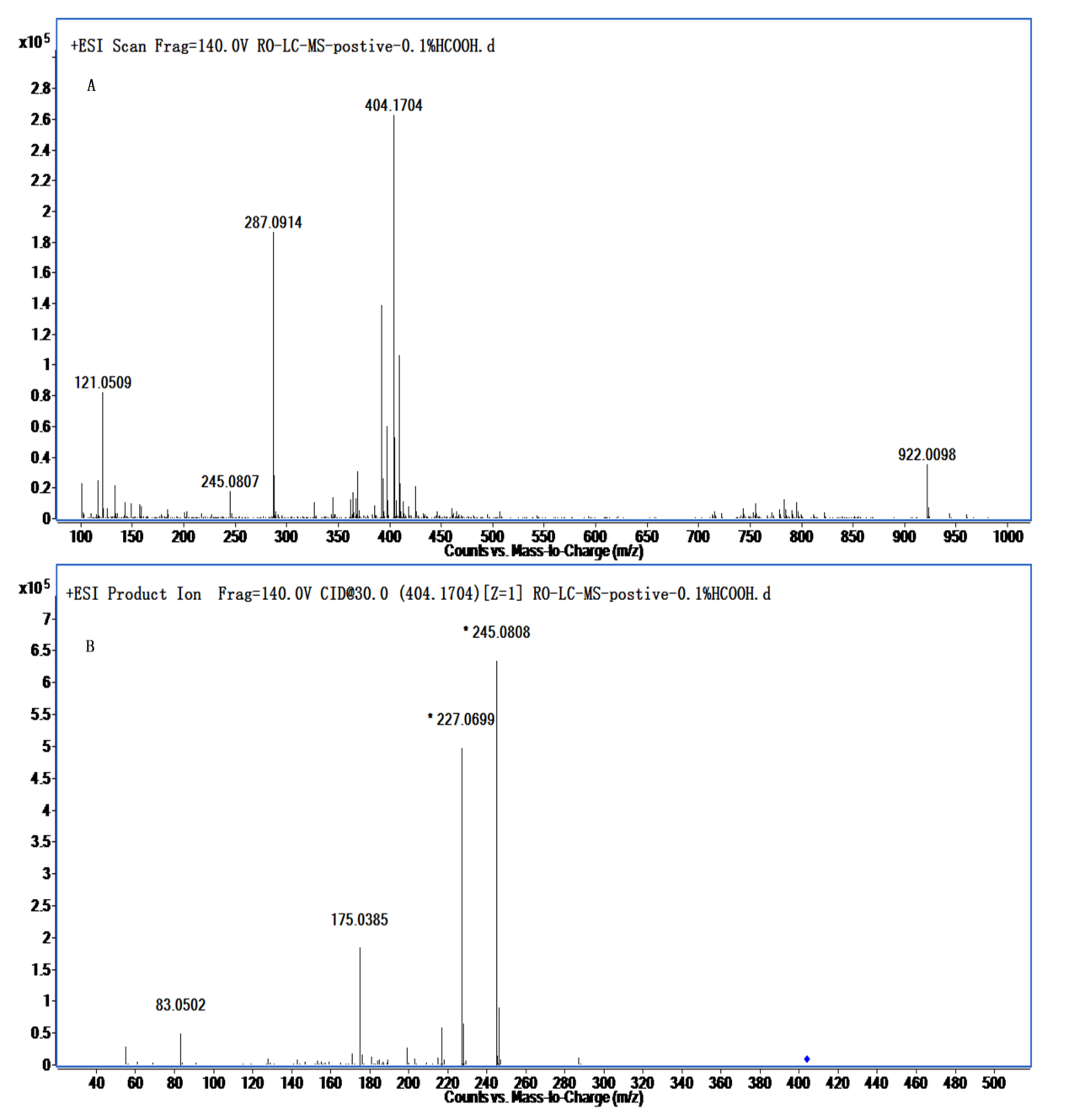


Figure S2 Primary scan (A) and secondary fragments (B) of (+)-praeruptorin A.


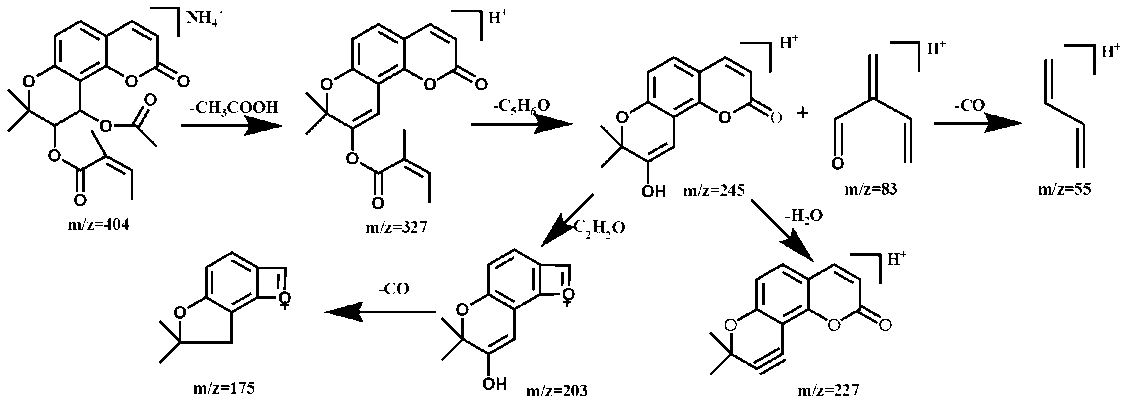


Figure S3 The deduced [fragmentation pathway](http://dict.cnki.net/dict_result.aspx?searchword=%e8%b4%a8%e8%b0%b1%e8%a3%82%e8%a7%a3%e8%a7%84%e5%be%8b&tjType=sentence&style=&t=fragmentation+regularities) of (+)-praeruptorin A.


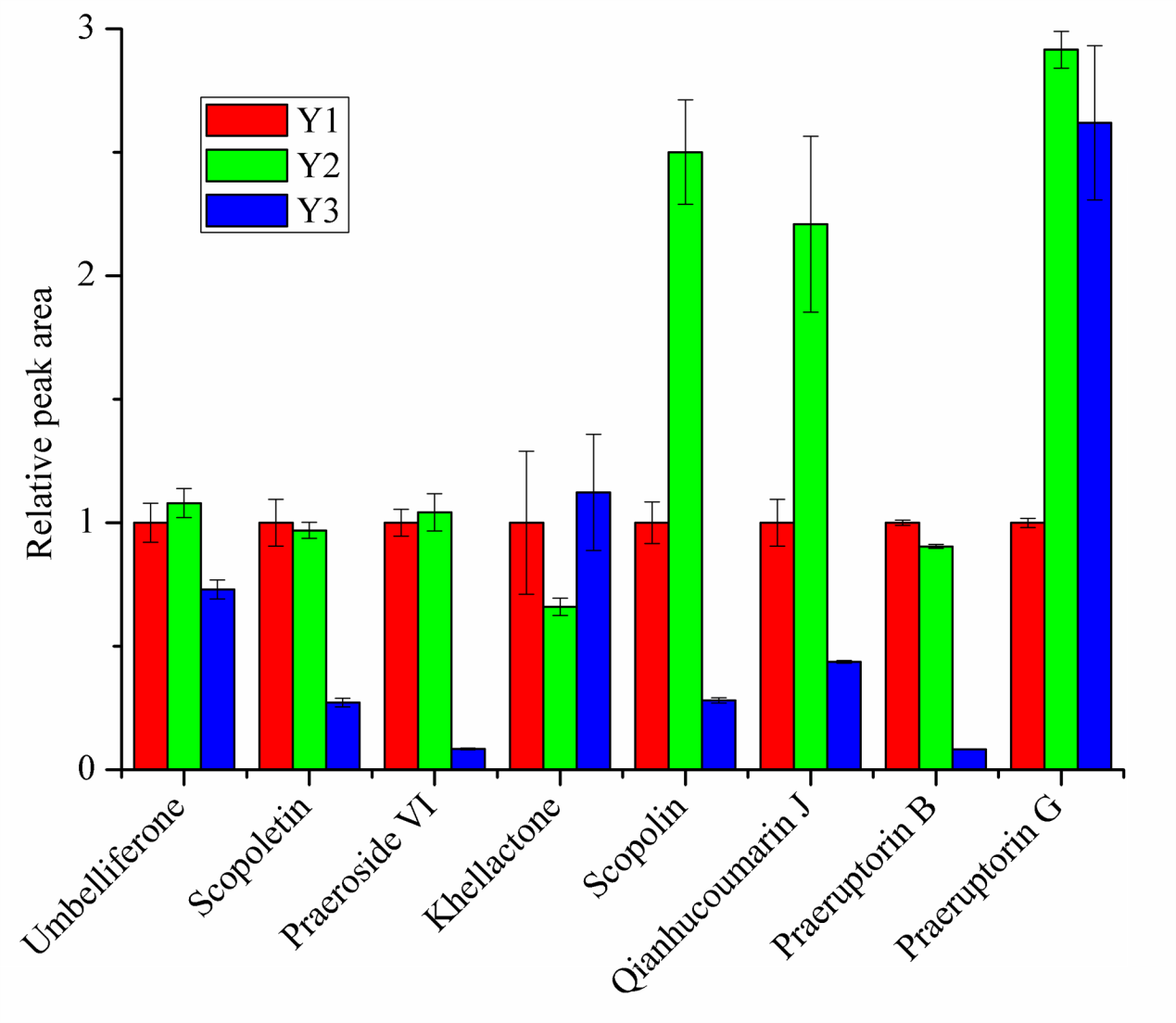


Figure S4 Contents variation of marker compounds.

Y1, Y2, Y3 represent one, two and three year after planting, respectively. For relative quantitative, the content of Y1was set as reference in each group, each bar represents the mean of triplicate experiments±standard deviation (SD).
